# Retaining Patients with Drug-Resistant Tuberculosis on Treatment During the COVID-19 Pandemic — Dharavi, Mumbai, India, 2020–2022

**DOI:** 10.15585/mmwr.mm7212a2

**Published:** 2023-03-24

**Authors:** Mangala D. Gomare, Sampada Bhide, Rajesh Deshmukh, Satish Kaipilyawar, Varsha Puri, Patrick K. Moonan, Dilip K. Khetade, Melissa Nyendak, Vijay Yeldandi, Jonathan P. Smith, James L. Tobias, Anand Date, Rajendra Joshi, Ravinder Kumar, Christine S. Ho

**Affiliations:** ^1^Brihanmumbai Municipal Corporation, Mumbai, India; ^2^Society for Health Allied Research and Education (SHARE) India, Hyderabad, India; ^3^Division of Global HIV and TB Response, Center for Global Health, CDC; ^4^Central TB Division, Ministry of Health and Family Welfare, Government of India, New Delhi, India.

Mumbai, India’s second largest city, has one of the highest prevalences of drug-resistant tuberculosis* (DRTB) in the world. Treatment for DRTB takes longer and is more complicated than treatment for drug-susceptible tuberculosis (TB). Approximately 300 persons receive a new DRTB diagnosis each year in Mumbai’s Dharavi slum^†^; historically, fewer than one half of these patients complete DRTB treatment. As nationwide restrictions to mitigate the COVID-19 pandemic were implemented, a program to facilitate uninterrupted DRTB care for patients receiving treatment was also implemented. A comprehensive tool and risk assessment provided support to DRTB patients and linked those who relocated outside of Dharavi during the pandemic to DRTB care at their destination. During May 2020–September 2022, a total of 973 persons received DRTB treatment in Dharavi, including 255 (26%) who relocated during treatment. Overall, 25 (3%) DRTB patients were lost to follow-up, a rate substantially lower than the rate before the pandemic (18%). Proactive planning and implementation of simple tools retained patients on treatment during periods of travel restrictions and relocations, improving programmatic outcomes. This approach might aid public health programs serving migrant populations or patients receiving treatment for DRTB during public health emergencies.

Mumbai, the capital of the state of Maharashtra, is India’s second most populous city. Within Mumbai, the Dharavi slum is the largest slum in Asia and one of the most densely populated areas in the world (1 million persons in 0.8 square miles [approximately 2.1 sq km]) and is a temporary home to persons seeking informal employment from across India.^§^ In 2019, Dharavi reported 265 DRTB patients, one of the highest concentrations of DRTB patients in the world; however, fewer than one half successfully finished treatment^¶^ (*1*,*2*). Low DRTB treatment completion is likely the consequence of the complexity of treating DRTB compared with that of treating drug-susceptible TB, including longer treatment durations, need for second-line drug regimens, more frequent drug-related adverse events, a higher prevalence of treatment relapse, and higher mortality (*3*).

In response to the COVID-19 pandemic in India, a series of government-enforced nationwide travel restrictions limited local, intrastate, and interstate movement during March 23–May 31, 2020, and January 27–April 30, 2021. Because of the large number of COVID-19 cases in Maharashtra, the state government extended these restrictions until June 15, 2021, during which time, movement was periodically allowed. During impending movement restrictions, many labor migrants from Dharavi, lacking a stable source of income, relocated to their permanent residences in India, traveling by foot, train, or bus (*4*).

Brihanmumbai Municipal Corporation (the governing civic body of Mumbai), CDC, and Society for Health Allied Research and Education (SHARE) India (a not-for-profit organization) implemented a public health intervention embedded within existing programmatic TB services to improve treatment outcomes and prevent treatment interruptions and migration-associated losses to follow-up. First, to establish and maintain care for DRTB patients** throughout the pandemic, a comprehensive risk assessment tool was developed that collected addresses (including permanent residence), telephone numbers of family members and close contacts, as well as potential travel routes. Destination sites and transit routes were mapped using collected information on common modes of travel. DRTB patients planning to relocate could apprise the project field coordinators of their plans during routine field encounters and make necessary preparations to continue treatment at their destination. Next, trained field coordinators, working with family members, governmental and nongovernmental organizations, community health workers, and district TB officers, used standard operating procedures to implement a series of interventions that included frequent patient contacting, active adverse events monitoring, and prompt attention to patients’ concerns to ensure continuity of care during and after relocation. For patients remaining within Dharavi, these interventions, implemented by field coordinators (who were exempted from travel restrictions), provided health services that included making telephone calls, home visits, treatment adherence counseling, and guidance for persons considering migration. For patients who had migrated, field coordinators informed the Dharavi TB program and telephoned patients and their destination TB programs to coordinate care. Visits and calls occurred every 2 weeks for the first 2 months and then monthly until treatment was completed. Routine TB services, which relied on patients coming to the TB clinic monthly and self-reporting treatment concerns and adverse events, were disrupted during the pandemic because of staff member reassignments and shortages. In response to the disruption, field coordinators visited patient homes to proactively monitor progress based on the schedule in the package of interventions (Box) and provided a 3-month supply of medications to patients for self-administration. In addition, the program connected persons to supplemental nutritional and monetary aid from governmental and nongovernmental programs. Address and travel information collected as part of the risk assessments aided in the identification of potential migrants. Migrants leaving Dharavi were provided a 1-month supply of medication to cover the potential travel period, and patients were connected with destination TB programs for continuation of care. Field coordinators counseled those who had already migrated to restart treatment in coordination with the destination TB program staff members. Participation was voluntary, and all participants had privacy and confidentiality protections. CDC provided funding and technical support; SHARE India was the implementing partner and provided the field coordinators. This intervention was reviewed by CDC and was conducted consistent with applicable federal law and CDC policy.^††^

BOX **Package of interventions for drug-resistant tuberculosis patients — Dharavi, Mumbai, India, May 2020–September 2022**

**Treatment administration**

- Every 2 weeks for the first 2 months of treatment
- After the first 2 months, every month until the end of treatment

**Location of treatment**

- At patient’s home by telephone or in-person visit
- At TB unit or clinic

**Persons who administer treatment**

- In Dharavi
  - Dharavi district TB staff members
  - TB field coordinators
  - DRTB patients or their family members
- Outside of Dharavi (to patients who have traveled)
  - District TB staff members at the patient’s destination
  - DRTB patients or their family members

**Activities conducted by field coordinators and TB staff members**

- Collect contact information for permanent residence, family members, and relatives
- Counsel patient about treatment adherence
- Monitor for adverse events
- Link to food and monetary initiatives
- Connect migrating DRTB patients, Dharavi TB program, and TB program staff members at permanent residence

**Abbreviations:** DRTB = drug-resistant tuberculosis; TB = tuberculosis.

During May 2020–September 2022, a total of 1,007 persons registered for DRTB treatment in Dharavi, and 973 (97%) initiated treatment, including 743 patients starting new treatment, and 230 who were already on treatment. The average age of DRTB patients was 28 years (range = 4–88 years), and 541 (56%) were female. Overall, 255 (26%) persons with DRTB relocated during treatment (Table). Among those who relocated, 70 (27%) informed program staff members of a planned relocation, and the remaining 185 persons (73%) were discovered to have relocated through discussions with household members during household visits or telephone calls. Among patients who relocated, 185 (73%) returned to Dharavi by January 2023 (cyclical migrants),^§§^ and 70 (28%) permanently relocated (permanent migrants).^¶¶^ The 255 patients who relocated moved to 14 states and Union Territories in India. Migrants traveled a median of 662 miles (1,065 km) (range = 7.5–1,317 miles [12–2,120 km]). Relocation was more common during the periods of May–June 2020 and April–June 2021) (Figure).

**TABLE:** Treatment outcomes among persons with drug-resistant tuberculosis — Dharavi slum,* Mumbai, India, May 2020–September 2022

Treatment outcome	No. (%)^†^				
	DRTB patients	Nonmigrants	Migrants^§^		
			All	Cyclical^¶^	Permanent**
Receiving DRTB treatment as of January 25, 2023	536 (55.1)	432 (60.2)	104 (40.8)	68 (36.8)	36 (51.4)
Treatment success^††^	360 (37.0)	237 (33.0)	123 (48.2)	100 (54.1)	23 (32.9)
Died	52 (5.3)	38 (5.3)	14 (5.5)	7 (3.8)	7 (10.0)
Lost to follow-up**^§§^**	25 (2.6)	11 (1.5)	14 (5.5)	10 (5.4)	4 (5.7)
**Total**	**973 (100.0)**	**718 (73.8)^¶¶^**	**255 (26.2)^¶¶^**	185 (72.5)***	70 (27.5) ***

**Abbreviations:** DRTB = drug-resistant tuberculosis; TB = tuberculosis.

* Defined as “a compact settlement of at least 20 households with a collection of poorly built tenements, mostly of temporary nature, crowded together usually with inadequate sanitary and drinking water facilities in unhygienic conditions." https://mohua.gov.in/upload/uploadfiles/files/9Slum_Report_NBO(2).pdf

^†^ Column percentage.

^§^ Persons who moved away from their usual place of residence.

^¶^ Migrants who left Dharavi but returned and reinstated DRTB treatment under the Dharavi TB program. Cyclical migrants might have relocated and returned several times, but eventually remained under treatment by the Dharavi TB program.

** Migrants who transferred out of Dharavi. Permanent migrants might have relocated and returned to Dharavi several times, but ultimately left and transferred out.

^††^ Confirmation of microbiologic TB cure or TB treatment completion.

^§§^ Did not start TB treatment or treatment interrupted for ≥2 consecutive months.

^¶¶^ Among all DRTB patients.

*** Among all migrants.

**FIGURE:**
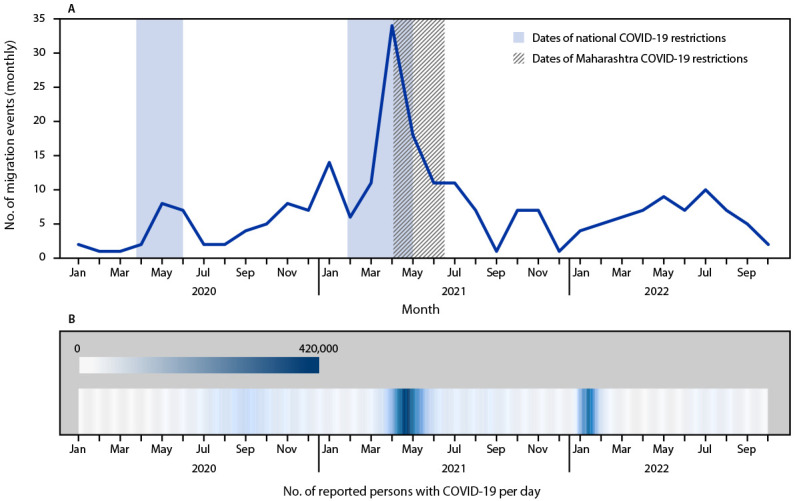
Number of monthly migration events of patients with drug-resistant tuberculosis, COVID-19 travel restrictions (A),*^,†^ and daily numbers of COVID-19 cases^§^ (B) — Dharavi, Mumbai, India, January 2020–October 17, 2022 * https://cdn.s3waas.gov.in/s3d18f655c3fce66ca401d5f38b48c89af/uploads/2020/03/2020032839.pdf † https://csmia.adaniairports.com/pdf/covid-19/20210530-gom-order-break-the-chain.pdf § https://covid19.who.int/ (Accessed February 13, 2023).

As of January 25, 2023, among all 973 Dharavi DRTB patients, 536 (55%) were receiving DRTB treatment; 360 (37%) had completed treatment; and 52 (5%) had died (Table). Overall, 540 (55%) patients reported 2,592 separate episodes of adverse events and were referred for medical evaluation; all but 101 (4%) episodes were resolved with medications for symptoms, adjustment in TB medications, or without intervention. All DRTB patients were signed up to receive governmental monetary support and referred for nongovernmental nutritional support. Only 25 (3%) of all Dharavi DRTB patients were lost to follow-up during the program’s implementation. Among the 255 patients who migrated, 104 (41%) were receiving treatment, 123 (48%) had completed treatment successfully, 14 (6%) had died, and 14 (6%) were lost to follow-up. The proportion of patients lost to follow-up was low among both cyclical (5%) and permanent migrants (6%), suggesting that the intervention measures were effective in both populations.

## Discussion

In countries such as India with high TB prevalence, clinics rely upon patients to collect their medicines monthly and self-report adverse events. By providing for the frequent contacting of patients, active monitoring of adverse events, and prompt addressing of concerns, this comprehensive package of interventions, integrated into routine programmatic care for DRTB treatment, facilitated continuity of care and improved treatment outcomes among patients from Dharavi during the COVID-19 pandemic. Nationwide, COVID-19 restrictions brought economic and logistic challenges to retaining DRTB patients in treatment. Implementation of the initial national travel restrictions in March 2020 resulted in many industries shutting down, leaving workers without wages. In anticipation of the restrictions, or after restrictions were lifted, many labor migrants traveled to and from Dharavi (*4*). During the peak of the COVID-19 pandemic, the Brihanmumbai Municipal Corporation TB program was concerned that contact with persons with DRTB would be lost during migration, or that they might default on treatment, resulting in untreated DRTB and potential transmission in communities throughout India. Working across states and sectors, a network of field coordinators and District TB Officers mitigated treatment interruption, monitored for adverse events, referred patients reporting adverse events, and connected DRTB patients to additional resources when needed. For this effort, staff members and field coordinators pivoted to use of telephones, to which most patients, their family members, or their neighbor had access. Recognizing that loss of wages was an important driving factor for the migration, field coordinators linked DRTB patients to additional nutritional and monetary support provided by government and nongovernment organizations. DRTB patients planning to relocate were encouraged to inform staff members during routine field encounters; only 3% of DRTB patients were lost to follow-up during the program’s implementation, a rate substantially lower than that before the COVID-19 pandemic (18%) (*2*).

Ensuring continuity of TB treatment is a priority during times of public health emergencies. Studies have described successful maintenance of TB treatment services for patients during natural disasters such as floods in Kerala, India (*5*), during hurricanes in the United States (*6*,*7*) and Puerto Rico (*8*), and after an earthquake in Haiti (*9*). In each of these circumstances, coordination across agencies and programs, accurate contact information, and dispensation of additional medication were necessary to ensure retention of TB patients. In the case of COVID-19, the scale and length of disruption was not localized to one geographic area; local and national travel restrictions added enormous challenges for provision of TB services.

The strategy implemented in Dharavi to retain DRTB patients for treatment was successful because it focused on addressing the difficulties of DRTB treatment for the patient by actively monitoring for treatment challenges. This was a simple but effective strategy, deployed under demanding circumstances; akin to other disruptive events, expanded coordination was needed to facilitate continuity of patient care (*5*–*9*).

The findings in this report are subject to at least two limitations. First, because many of the patient support activities occurred over the telephone, assessment of treatment adherence might have been overestimated. Second, strained health systems and staffing shortages meant that TB test results were delayed, which might have affected categorization of treatment outcomes. Thus, the proportion of patients that remained on DRTB treatment might be overestimated.

During public health emergencies, challenges to DRTB treatment completion are common, especially among persons who subsist on low wages and those without a social or financial safety net. The approach implemented in Dharavi has been adopted by the Brihanmumbai Municipal Corporation in other densely populated, poor urban settings to improve DRTB treatment and care and might aid public health programs that serve migrant populations or DRTB patients during public health emergencies.

**Summary**

**What is already known about this topic?**

Treatment for drug-resistant tuberculosis (DRTB) takes longer and is more complicated than treatment for drug-susceptible tuberculosis. The Dharavi slum in Mumbai, India has one of the highest concentrations of DRTB patients in the world. The COVID-19 pandemic disrupted TB care and treatment.

**What is added by this report?**

During the pandemic, many persons with DRTB in Dharavi relocated, threatening continuity of care. Patient-focused interventions facilitated successful treatment retention and improved programmatic outcomes.

**What are the implications for public health practice?**

Planning and implementation of simple tools helped to retain migrants on DRTB treatment during periods of COVID-19 restrictions and relocations; this approach might aid programs to serve persons on treatment for DRTB during public health emergencies, including migrant populations.

## Footnotes

* A *Mycobacterium tuberculosis* isolate that is resistant to isoniazid or rifampin; resistance to additional TB medications can also be present.

† Defined as “a compact settlement of at least 20 households with a collection of poorly built tenements, mostly of temporary nature, crowded together usually with inadequate sanitary and drinking water facilities in unhygienic conditions." https://mohua.gov.in/upload/uploadfiles/files/9Slum_Report_NBO(2).pdf

§
https://sra.gov.in/page/innerpage/about-drp.php

¶
https://tbcindia.gov.in/showfile.php?lid=3538

** Treatment for DRTB was in accordance with the India national programmatic guidelines for drug-resistant TB. https://tbcindia.gov.in/showfile.php?lid=3590

†† 45 C.F.R. part 46, 21 C.F.R. part 56; 42 U.S.C. Sect. 241(d); 5 U.S.C. Sect. 552a; 44 U.S.C. Sect. 3501 et seq.

§§ A migrant who left Dharavi but returned and reinstated DRTB treatment under the Dharavi TB program. A cyclical migrant might have relocated and returned several times, but eventually remained under treatment by the Dharavi TB program.

¶¶ A migrant who relocated from Dharavi and transferred out. Permanent migrants might have relocated and returned to Dharavi several times, but ultimately left and transferred out.
